# A Mendelian randomization study of the association between serum uric acid and osteoporosis risk

**DOI:** 10.3389/fendo.2024.1434602

**Published:** 2024-10-11

**Authors:** Heng Wu, Hairui Li, Xiao Dai, Yu Dai, Hao Liu, Shuang Xu, Jinbang Huang, Hao Chi, Song Wang

**Affiliations:** ^1^ Department of Orthopedics, the Affiliated Hospital of Southwest Medical University, Luzhou, China; ^2^ Clinical Medical College, Southwest Medical University, Luzhou, China

**Keywords:** serum uric acid, osteoporosis, genetic, casual effect, Mendelian randomization

## Abstract

**Background:**

The relationship between serum uric acid (SUA) and osteoporosis (OP) has yielded conflicting results in observational studies. This Mendelian randomization (MR) study aims to elucidate the causal association between SUA and OP.

**Methods:**

A two-sample MR analysis was conducted using summary-level data from genome-wide association studies (GWAS). Two sets of polygenic instruments strongly associated (p < 5 × 10^-8^) with SUA were extracted from the CKDGen consortium and UK biobank. Polygenic instruments associated with OP (p < 5 × 10^-8^) were derived from FinnGen biobank and UK biobank. Inverse variance weighting (IVW) was employed as the primary analysis method. Additionally, we utilized MR-Egger, weighted median, the simple mode method, and the weighted mode as complementary analyses. Cochran’s Q statistics were used to assess heterogeneity, with MR-Egger intercept testing and MR pleiotropy residual sum and outlier (MR-PRESSO) to examine horizontal pleiotropy. Sensitivity analysis was performed using the leave-one-out method.

**Results:**

The IVW analysis conducted across four groups confirms no significant causal relationship between SUA concentration and OP: UKB-UKB (OR: 1.001, 95% CI: 0.999-1.003, p=0.464), CKD-UKB (OR: 1.001, 95% CI: 0.999-1.003, p=0.349), UKB-Fin (OR: 0.934, 95% CI: 0.747-1.168, p=0.549), CKD-Fin (OR: 1.041, 95%CI: 0.934-1.161, p=0.470). Furthermore, additional four MR analyses corroborated these findings. Upon excluding all outliers identified by the MR-PRESSO test, no significant directional pleiotropy was observed, except for some data heterogeneity noted in the UKB-UKB group (Q=50.65, P=0.002). Additionally, a leave-one-out analysis indicated that no single SNP exerted undue influence on the results.

**Conclusion:**

This MR analysis provides convincing genetic evidence that there is no causal association between SUA and OP, SUA is unlikely to increase or reduce the risk of OP.

## 1 Introduction

Osteoporosis (OP) is a metabolic skeletal disease characterized by imbalances in bone homeostasis, resulting in decreased bone mass and deterioration of bone microarchitecture, thereby increasing the susceptibility to fragility fractures (1). With a global incidence rate of 18.3%, OP is highly prevalent and imposes a substantial economic burden, estimated at 6.5 trillion dollars in the US, Canada, and Europe alone (2, 3). Due to its high prevalence and associated disability, morbidity, and subsequent osteoporotic fractures, OP has become a serious public health problem (4–6).

Serum uric acid (SUA) is considered to end product of purine nucleotide degradation (7). SUA has two paradoxical functions in human body. In plasma, SUA exhibits antioxidant properties and offers a protective effect on bone metabolism by promoting bone formation and inhibiting bone resorption. Conversely, intracellularly, SUA acts as a pro-oxidant, leading to increased inflammatory and oxidative stress levels that contribute to bone loss by disrupting osteoclast and osteoblast activities (8, 9). Numerous observational studies have reported inconsistent findings regarding the relationship between SUA and OP, suggesting that SUA could potentially have a positive, neutral, or negative impact on the development of OP (10–14). These conflicting findings underscore the challenges in elucidating the causal association between SUA and OP, particularly in the presence of unmeasured confounders. Additionally, the potential for reverse causality and regression dilution bias further complicates efforts to establish causation.

Mendelian randomization (MR) analysis is a powerful statistical approach in genetic epidemiology, utilizing genetic variants as instrumental variables (IVs) to estimate causal associations between risk factors and disease outcomes (15, 16). These genetic variants are determined before embryo formation, making them impervious to confounding factors and acquired diseases (17). Therefore, the MR can avoid the limitations of observational studies including confounding, reverse causality and regression dilution bias (18). MR typically uses single-nucleotide polymorphisms (SNPs) as IVs, often sourced from genome-wide association studies (GWAS) summarizing associations of IVs with various traits (19). To date, MR has not yet been used to elucidate the causal association of SUA on OP. In this study, we employ a two-sample MR analysis to determine whether SUA is causally linked to OP, which providing a novel approach to investigating the causal relationship between SUA on OP. While observational studies have shown inconsistent results, MR helps clarify potential causal effects by reducing biases and confounding factors. This is crucial for advancing our understanding of SUA’s role in bone health and could influence future prevention and treatment strategies for osteoporosis, a condition with significant public health implications. By addressing existing gaps in the literature, our study offers new insights that could guide future research and clinical practices.

## 2 Methods

### 2.1 Research design

Through literature retrieval, we found multiple GWAS databases for both SUA concentration and OP. To investigate the relationship between SUA concentration and OP more comprehensively, we selected two databases each for exposure and outcome GWAS (SUA concentration: Chronic Kidney Disease Genetics Consortium [CKDGen], UK Biobank [UKB]; OP: FinnGen Release 10, UK Biobank), and conducted a 2*2 combination analysis between exposure and outcome. This approach yielded four result groups: CKD-Fin, CKD-UKB, UKB-Fin, and UKB-UKB, enabling a more comprehensive study of the relationship between SUA concentration and OP.

### 2.2 Data sources

To investigate the causal relationship between SUA and OP, we selected SNPs from GWAS databases as IVs. These databases are publicly accessible, thus requiring no additional ethical approval.

Serum urate GWAS data were sourced from the Chronic Kidney Disease Genetics Consortium (CKD Gen) and the UK Biobank. CKDGen conducted the latest GWAS on urate levels of 288,649 European participants (20). To validate the robustness of our findings and minimize bias, we also utilized urate level data from 343,836 participants in the UK Biobank, which can be accessed at https://gwas.mrcieu.ac.uk/datasets (ID: ebi-a-GCST90018977).

The osteoporosis GWAS data were obtained from the FinnGen Release 10 database (https://www.finngen.fi/en), comprising 8,017 cases and 391,037 controls from European populations. This dataset utilized ICD-10, ICD-9, and ICD-8 coding for osteoporosis diagnosis (21). Another osteoporosis dataset consisted of 484,598 participants from the UK Biobank (UKB), accessible for download at https://gwas.mrcieu.ac.uk/datasets (ID: ebi-a-GCST90038656).

### 2.3 Instrumental variable selection

**Figure 1:**
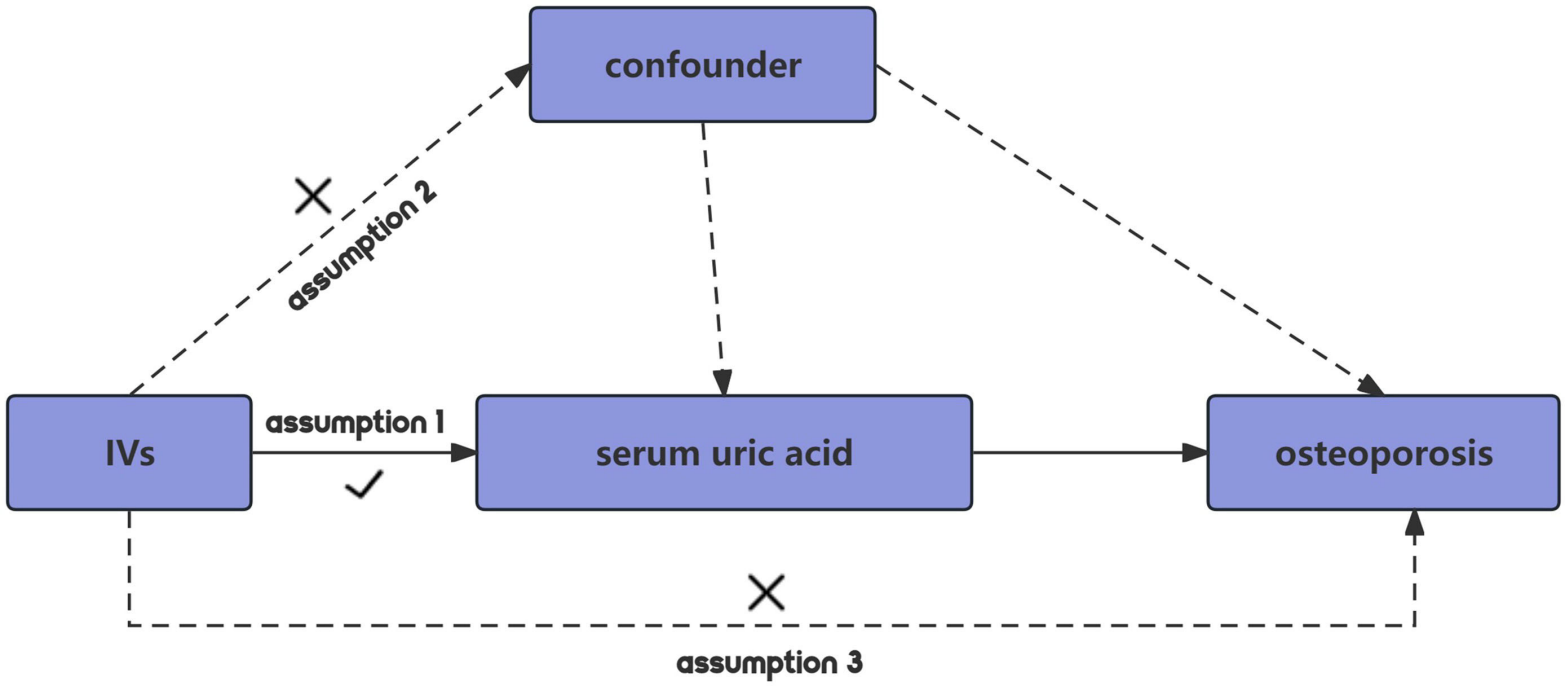
Schematic diagram of MR.

MR employing SNPs as IVs should adhere to three core assumptions to minimize bias in the outcomes (22). Firstly, the relevance assumption dictates that IVs must exhibit a strong correlation with the exposure. Secondly, the independence assumption stipulates that genetic variations must be independent of unmeasured confounders that could affect the exposure-outcome association. Lastly, the exclusion restriction assumption posits that the IV influences the outcome solely through its association with the exposure, thus maximizing the reduction of pleiotropic effects (**Figure 1**).

**Figure 2:**
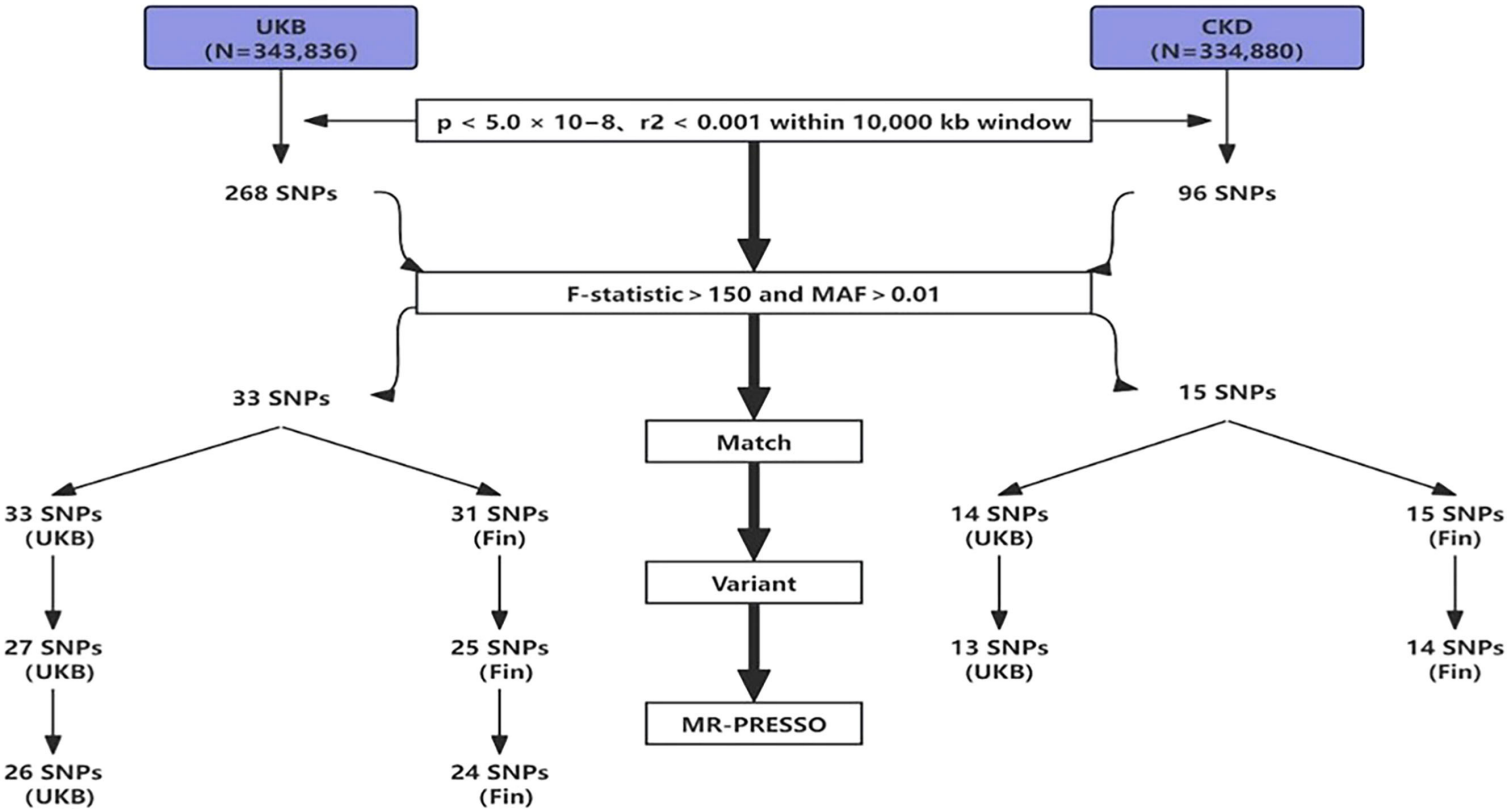
The flowchart of IVs selection.

To mitigate potential interference from linkage disequilibrium between SNPs, ensuring the accuracy and reliability of causal inference between SUA and OP, we implemented several restrictive measures in SNPs selection. Initially, using a threshold of p<5.0×10^-8^, we identified genome-wide significant SNPs for uric acid, followed by exclusion of SNPs in linkage disequilibrium using predefined parameters (r2<0.001 within a 10,000kb window), ensuring the independence of the selected IVs (23). Furthermore, we evaluated the strength of association between each IV and exposure, excluding weak IVs. The F-statistic for each IV was calculated using the formula: F=Beta2/SE2 (where Beta represents the estimated effect of the allele on exposure, and SE denotes standard error (24). To account for potential genetic confounding or measurement error, we utilized the F-statistic to exclude IVs, as higher F-statistics indicate stronger associations (25, 26). Combining the number of IVs, we ultimately set F>150 as the exclusion criterion. Finally, during variant harmonization, SNPs that could not match the outcome dataset and palindromic SNPs were excluded. The flowchart of IVs selection is shown below (**Figure 2**).

### 2.4 Statistical analysis

In our study, statistical analyses were performed using R software (version 4.3.2) and the Two-Sample MR package (version 0.5.9), as well as the MR-PRESSO software package. To explore the causal relationship between SUA and OP, we employed five different MR analysis methods: inverse variance-weighted (IVW) method, MR-Egger, weighted median, weighted mode, and simple mode, with IVW being the primary method. In the absence of directional pleiotropy, IVW provides cumulative causal estimates based on the Wald ratio derived from each IV (19). The MR-Egger method yields relatively robust estimates, even in the presence of all SNPs being invalid, by introducing an intercept term and regression slope on top of IVW, thereby detecting and adjusting for horizontal pleiotropy (27). Weighted median provides stable results even when over 50% of the weight comes from invalid IVs, while weighted mode yields robust overall causal estimates when most similar individual estimates come from valid IVs (28, 29). Finally, we employed the MR-PRESSO method to provide effective estimates in the presence of horizontal pleiotropy. MR-PRESSO identifies genetic variants significantly impacting causal estimates, removes interference from outliers, and offers corrected results after outlier removal (30).

Given that IVW method may not adequately address confounding between SNPs, it was imperative to assess heterogeneity before IVW application. We assessed heterogeneity among IVs using Cochrane’s Q statistic, employing a leave-one-out sensitivity test, sequentially excluding one IV at a time to assess the stability of MR results (31).

## 3 Results

### 3.1 Genetic instrumental variable selection

We processed the SNP data from the exposure group downloaded from the GWAS database as follows: (1) SNPs not meeting the criteria were excluded using a threshold of p<5.0×10^-8^ and an r^2<0.001 within a 10,000kb window (23); (2) SNPs with F>150 and MAF>0.01 were selected for high association strength; (3) The selected exposure group SNPs were matched with outcome group SNPs to obtain shared SNPs between exposure and outcome; (4) Shared SNPs were subjected to variant harmonization to remove palindromic sequences; (5) The obtained SNP data underwent MR-PRESSO analysis to remove SNPs with outliers. After these 5 steps, SNPs were included in the final analysis. In total, we included 26 SNPs (UKB-UKB), 24 SNPs (UKB-Fin), 13 SNPs (CKD-UKB), and 14 SNPs (CKD-Fin). The specific selection process is illustrated in the flowchart (**Figure 2**).

### 3.2 Casual relationship between SUA concentration and OP

**Figure 3:**
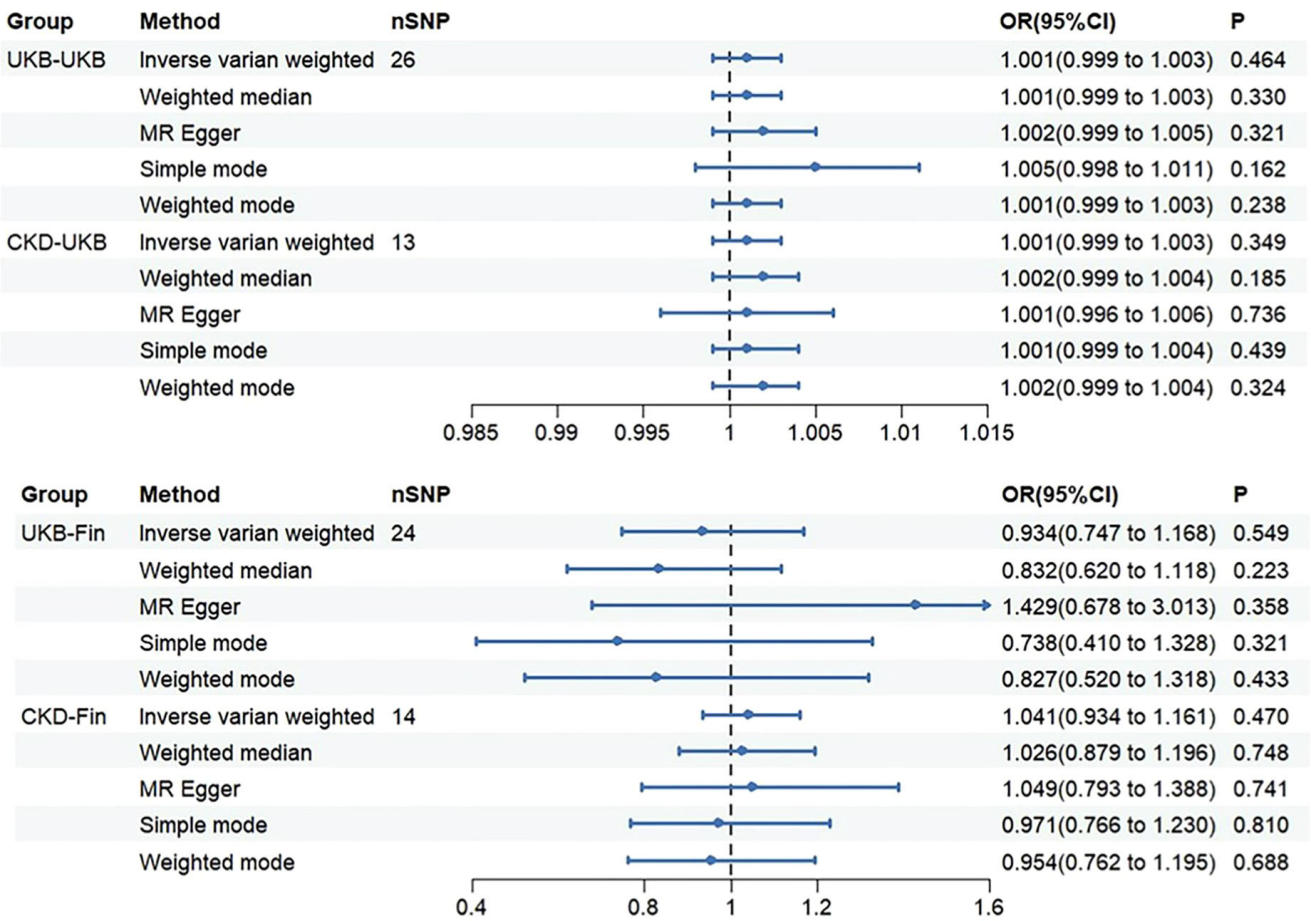
The OR (odds ratio) value and 95%CI (confidence interval) of IVW, MR-Egger, weighted median, weighted mode, and simple mode in each group.

**Figure 4:**
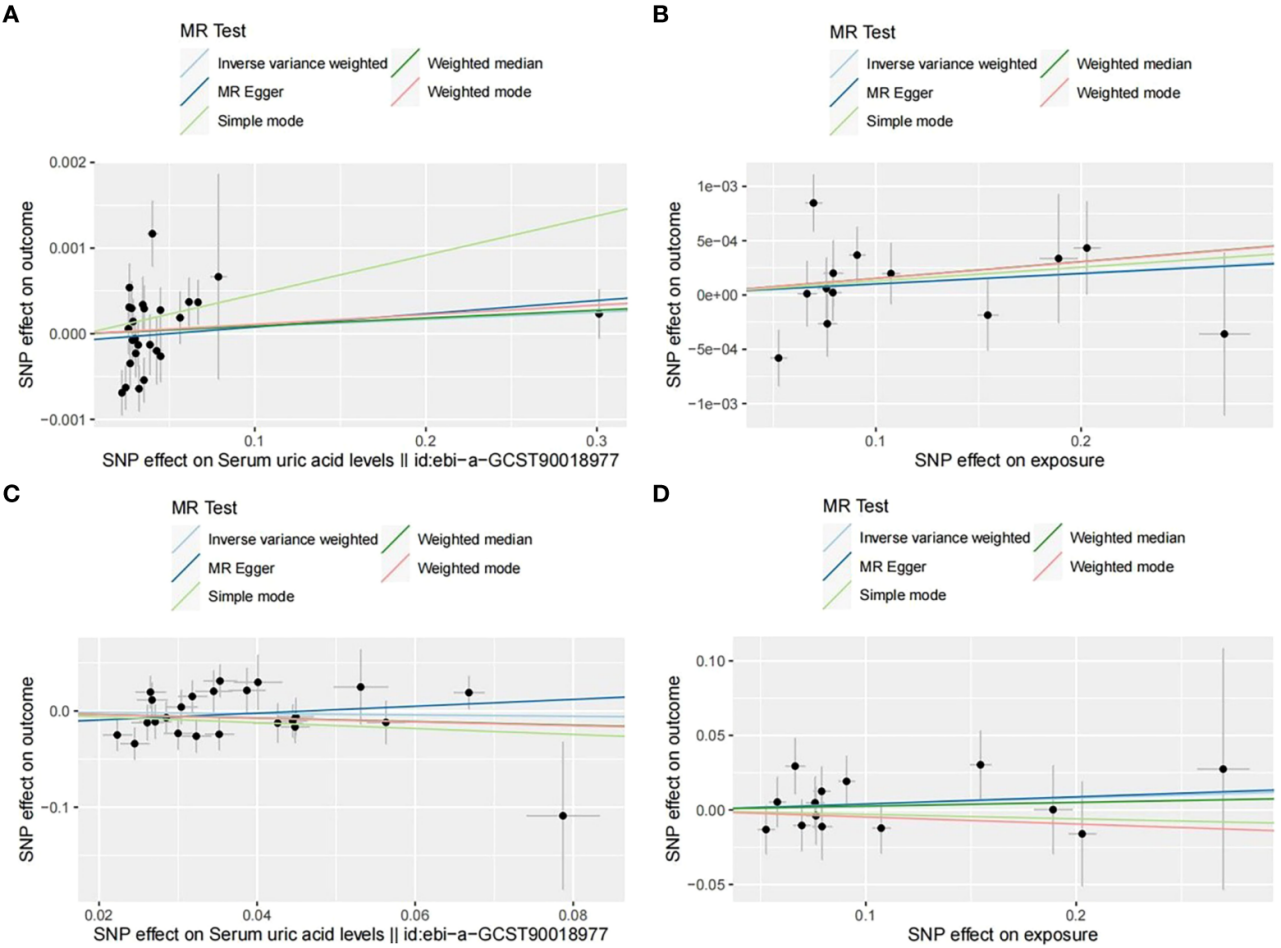
Scatter plots of each group. **(A)** UKB-UKB. **(B)** CKD-UKB. **(C)** UKB-Fin. **(D)** CKD-Fin.

Through analysis of data from each group, we found no statistically significant causal relationship between SUA concentration and OP, as illustrated in the **Figure 3**. Detailed data are presented below. Scatter plot (**Figure 4**) is provided below.

#### 3.2.1 UKB-UKB

Through Cochran’s Q test, we identified significant heterogeneity (Q=60.137, p=1.61e-04). Therefore, for this MR analysis, we primarily employed a multiplicative random effects model. The global test of MR-PRESSO reported an outlier (rs1260326, RSSobs=6.563e-07, p<0.027). After removing this outlier, based on the inverse variance weighted method (OR: 1.001, 95% CI: 0.999-1.003, p=0.464), we found no statistically significant positive causal relationship between SUA concentration and OP. Similar results were observed with the weighted median method (OR: 1.001, 95% CI: 0.999-1.003, p=0.330). Subsequent analyses using the MR-Egger method (OR: 1.002, 95% CI: 0.999-1.005, p=0.321), simple mode (OR: 1.005, 95% CI: 0.998-1.011, p=0.162), and weighted mode (OR: 1.001, 95% CI: 0.999-1.003, p=0.238) also showed no significant causal relationship.

#### 3.2.2 CKD-UKB

We initially conducted Cochran’s Q test on the data and found no evidence of heterogeneity (Q=19.979, p=0.067). Therefore, for this set of MR analyses, we employed a fixed-effects model. IVW analysis revealed no significant causal relationship between SUA concentration and OP (OR: 1.001, 95% CI: 0.999-1.003, p=0.349). Subsequent analyses using the weighted median (OR: 1.002, 95% CI: 0.999-1.004, p=0.185), MR-Egger method (OR: 1.001, 95% CI: 0.996-1.006, p=0.736), simple mode (OR: 1.001, 95% CI: 0.999-1.004, p=0.439), and weighted mode (OR: 1.002, 95% CI: 0.999-1.004, p=0.324) did not reveal any significant causal relationship between SUA concentration and OP.

#### 3.2.3 UKB-Fin

We observed significant heterogeneity through Cochran’s Q test (Q=39.584, p=0.024). Hence, for this set of MR analyses, we also employed a multiplicative random effects model. MR-PRESSO global test identified an outlier (rs589852, RSSobs=3.014e-03, p=0.025). After removing this outlier, we utilized the IVW analysis method as our primary approach (OR: 0.934, 95% CI: 0.747-1.168, p=0.549). Interestingly, contrasting the first group, we obtained a reverse result, yet similarly, no statistically significant causal relationship between SUA concentration and OP was evident. The weighted median method similarly showed no statistically significant causal relationship (OR: 0.832, 95% CI: 0.620-1.118, p=0.223). Subsequently, we employed three methods for analysis: MR-Egger method (OR: 1.429, 95% CI: 0.678-3.013, p=0.358), simple mode (OR: 0.738, 95% CI: 0.410-1.328, p=0.321), and weighted mode (OR: 0.827, 95% CI: 0.520-1.318, p=0.433). Notably, in this analysis, MR-Egger method showed a direction inconsistent with the other four methods, which will be discussed further. However, regardless of directionality, none of these methods exhibited a clear causal relationship.

#### 3.2.4 CKD-Fin

Consistent with the previous group, we initially performed Cochran’s Q test, which indicated no evidence of heterogeneity (Q=7.926, p=0.848). Therefore, we employed a fixed-effects model for the MR analysis in this group. Based on the IVW analysis, we found no significant causal relationship between SUA concentration and OP (OR: 1.041, 95%CI: 0.934-1.161, p=0.470). Subsequently, we utilized four methods for the analysis: weighted median (OR: 1.026, 95%CI: 0.879-1.196, P=0.748), MR-Egger (OR: 1.049, 95%CI: 0.793-1.388, P=0.741), simple mode (OR: 0.971, 95%CI: 0.766-1.230, P=0.810), and weighted mode (OR: 0.954, 95%CI: 0.762-1.195, P=0.688). All results consistently indicated no significant causal relationship between SUA concentration and OP. Worth noting, however, is that the simple and weighted modes showed the same directional inconsistency as the second group, which will be discussed in the subsequent sections.

### 3.3 Sensitivity analysis

**Figure 5:**
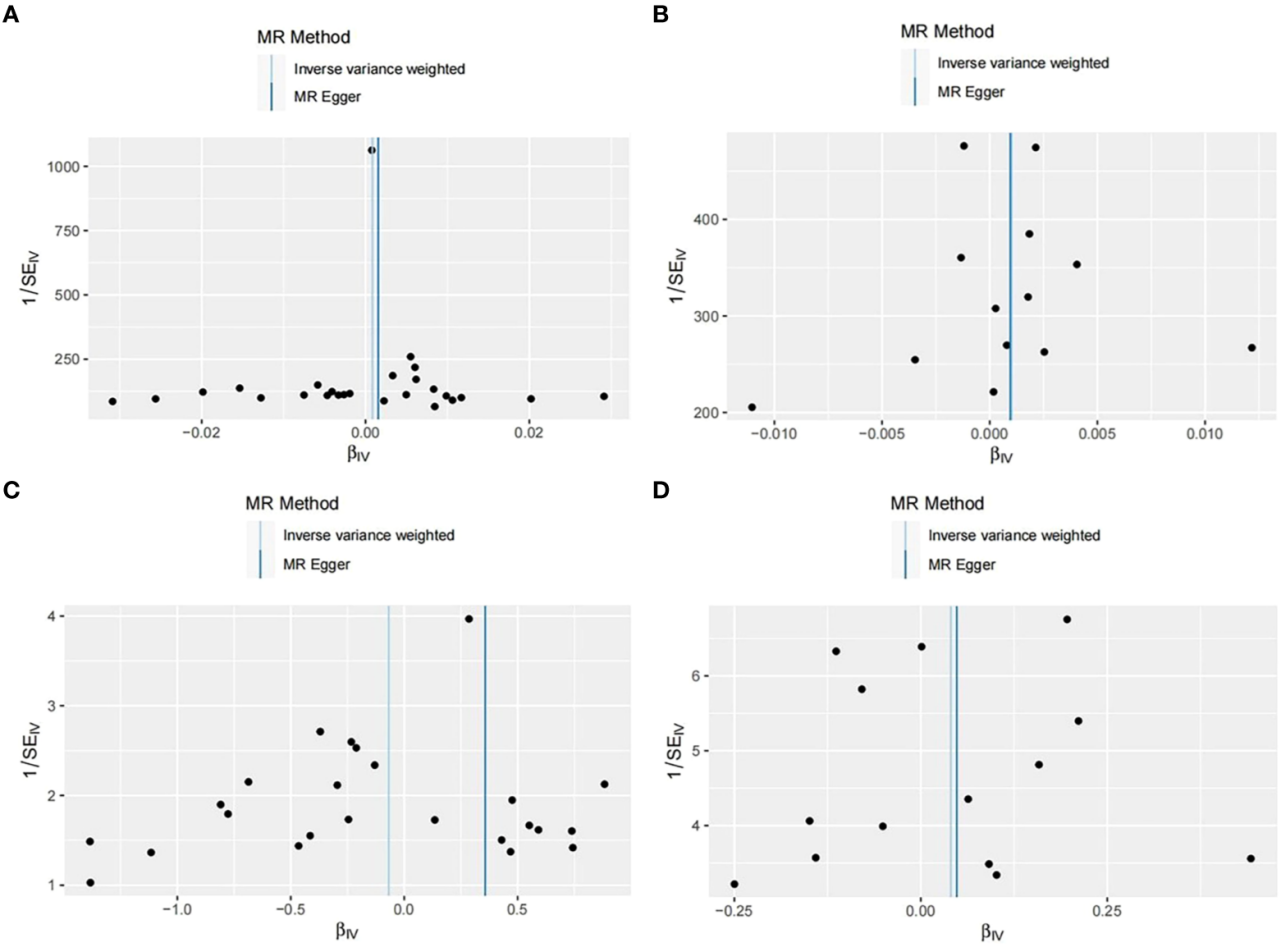
Funnel plots of four groups. **(A)** UKB-UKB. **(B)** CKD-UKB. **(C)** UKB-Fin. **(D)** CKD-Fin.

In our study, we initially assessed the heterogeneity of the SNPs included in the final MR analysis using the Q statistic. The heterogeneity results were as follows: UKB-UKB group (Q=50.65, P=0.002), CKD-UKB group (Q=19.979, p=0.067), UKB-Fin group (Q=28.62, p=0.193), CKD-Fin group (Q=7.926, p=0.848). Funnel plot (**Figure 5**) is provided below. Except for the UKB-UKB group, which showed some heterogeneity, the other groups did not exhibit significant heterogeneity.

Subsequently, we conducted horizontal pleiotropy tests using both MR-Egger intercept test and MR-PRESSO. The results of the MR-Egger intercept test for each group were as follows: UKB-UKB group (Egger intercept = -7.58e-05, p=0.484), CKD-UKB group (Egger intercept = 6.80e-06, p=0.981), UKB-Fin group (Egger intercept = -0.017, p=0.254), CKD-Fin group (Egger intercept = -8.17e-04, p=0.953). MR-PRESSO test did not detect any outliers, confirming the absence of horizontal pleiotropy in each group.

**Figure 6:**
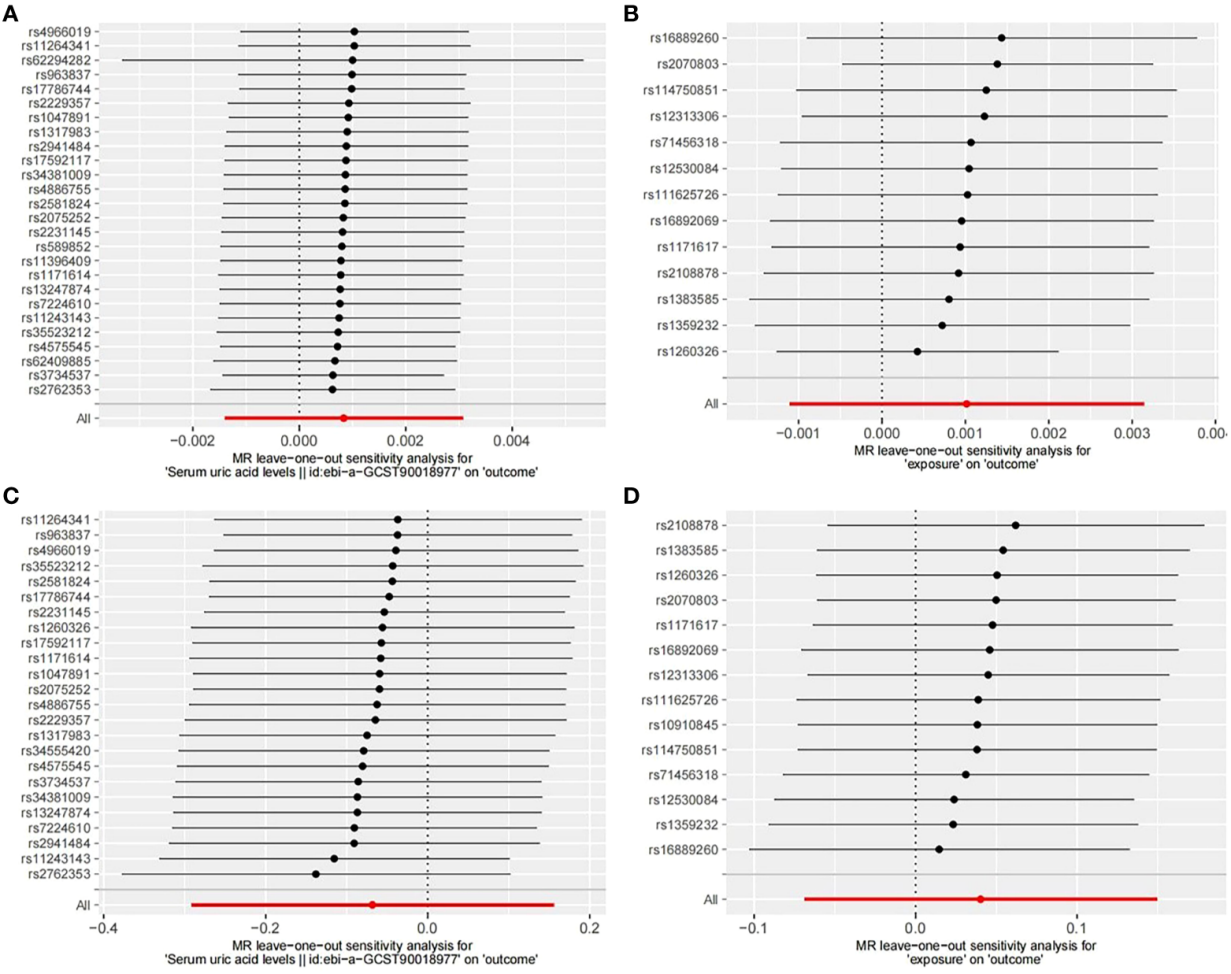
Leave-one-out sensitive analysis of SNPs associated with SUA and OP. **(A)** UKB-UKB. **(B)** CKD-UKB. **(C)** UKB-Fin. **(D)** CKD-Fin.

Finally, we conducted a leave-one-out sensitivity analysis (**Figure 6**) by sequentially removing one SNP at a time, recalculating the causal effect of the remaining SNPs, and observing whether the results changed with the removal of each SNP. The sensitivity analysis further affirmed the reliability of our study results (**Table 1**).

**Table 1:** Sensitivity analysis of MR analyses.

Group	nSNP	Heterogeneity test		MR-Egger pleiotropy test		MR-PRESSO global pleiotropy test	
		Q	p-Value	Intercept	p-Value	RSSobs	p-Value
UKB-UKB	26	50.65	0.002	-7.58E-05	0.484	51.683	0.026
CKD-UKB	13	19.98	0.067	6.80E-06	0.981	22.474	0.108
UKB-Fin	24	28.62	0.193	-0.017	0.254	31.313	0.210
CKD-Fin	14	7.93	0.848	-8.17E-04	0.953	9.326	0.858

nSNP, number of SNP.

## 4 Discussion

In this study, we designed a two-sample MR analysis to detect the causal relationship of SUA and OP. Our MR analysis results demonstrate that no genetic evidence was found to support the causal association between SUA and OP, based on data from the largest published GWAS.

Most clinical observational studies found no consistent causal relationship of SUA on OP (10–14). A large Asian cohort study involving 119,037 participants revealed through logistic regression analysis that elevated SUA levels were associated with a reduced risk of OP in females but not in males (10). Similarly, a retrospective study encompassing 173,209 participants from Korea showed a negative correlation between high SUA levels and OP risk (11). However, a cross-sectional study of US males found no discernible causal link between SUA and OP (12). Further complicating matters is a study involving the US general population and rats with experimental hyperuricemia which found no significant relationship between SUA and OP in either group (13). Conversely, a prospective cohort study confirmed that SUA was associated with an increased risk of hip fractures in men (14). Uric acid metabolism can affect normal bone metabolism through numerous pathways, which can increase or decrease the risk of OP. Oxidative stress is recognized as a pivotal factor in the pathogenesis of OP, as it promotes osteoclast formation, triggers apoptosis in osteoblasts and osteocytes, and inhibits osteoblast differentiation and activity (32). Consumption of high-antioxidant whole plant foods or adopting an antioxidant-rich lifestyle can enhance bone mineral density (BMD) by curbing reactive oxygen species (ROS) production (1, 32). SUA acts as an antioxidant, countering different oxidants like superoxide anions, hydrogen radicals, and peroxynitrite, thereby offering protective effects on bone metabolism. Conversely, as a pro-oxidant, SUA can contribute to bone loss by elevating inflammatory and oxidative stress levels (8, 9). Additionally, SUA can disrupt vitamin D metabolism and increase serum parathyroid hormone (PTH) concentration, exacerbating bone loss (8). However, observational studies on this matter have inherent limitations, such as methodological flaws, small sample sizes, selection bias, and inadequate adjustment for confounding factors. As a result, a definitive causal relationship between SUA and OP cannot be established solely based on these studies.

MR is a method used in human genetic research that leverages genetic variations as IVs for causal inference. In our study, we investigated the relationship between SUA concentration and OP by employing genetic variations as IVs. MR enables the control of non-heritable environmental confounders’ influence on OP and mitigates directional causation biases. Additionally, we utilized the F-statistic as a criterion for data selection, whereby a higher F-value indicates a stronger association between SNPs and SUA concentration. SNPs with an F-value greater than 150 were retained, while weakly associated IVs were removed to exclude potential bias (33). We also assessed horizontal pleiotropy, which occurs when genetic variations directly influence other traits or outcomes. The presence of horizontal pleiotropy would challenge the assumption that IVs established in this MR analysis solely affect OP through SUA concentration. We conducted four sets of MR analyses, incorporating 26, 24, 13, and 14 SNPs, respectively, with the final results indicating no significant causal relationship between SUA concentration and OP. Notably, our multiple MR analyses yielded consistent outcomes. We followed relevant MR guidelines to mitigate biases, including sourcing genetic variations from different datasets as supplementary IVs.

In sensitivity analyses, we assessed both horizontal pleiotropy and heterogeneity. For horizontal pleiotropy, we primarily employed MR-Egger intercept tests and MR-PRESSO. Across all MR-Egger intercept tests, non-zero intercepts with p>0.05 indicated no evidence of horizontal pleiotropy. Furthermore, MR-PRESSO analyses identified outliers in the UKB-UKB (rs1260326, RSSobs=6.563e-07, p<0.027) and UKB-Fin (rs589852, RSSobs=3.014e-03, p=0.025) groups, potentially impacting causal inference. After removing outliers, subsequent MR-PRESSO analyses revealed no evident horizontal pleiotropy, leading to the inclusion of the remaining SNPs for analysis. Regarding heterogeneity, we utilized Cochran’s Q statistic to test for differences among IVs, with results indicating no significant heterogeneity except for the UKB-UKB group. While heterogeneity can arise from various sources such as different analytical platforms, experiments, or populations, its presence can affect MR analysis outcomes. To address heterogeneity, we employed random-effects models to reduce its impact. Despite Cochrane’s Q statistic indicating heterogeneity in the UKB-UKB group, none of the MR analyses supported significant causal relationships. Finally, we conducted leave-one-out sensitivity analyses, which confirmed the reliability of our study, as all results aligned on the same side of zero.

This study has some limitations. Firstly, despite conducting multiple MR analyses with independently acquired data, potential sample overlap cannot be ruled out. Secondly, the study primarily utilized GWAS databases of European populations, limiting the generalizability of findings to other populations. Thirdly, with a focus on assessing and interpreting the overall effects between SUA and OP to ensure the consistency and reliability of the conclusion, we did not conduct subgroup analysis. However, exploring potential effect heterogeneity or modifiers could offer deeper insights into our findings in the future.

## 5 Conclusion

This MR analysis provides convincing genetic evidence that there is no causal association between SUA and OP, SUA is unlikely to increase or reduce the risk of OP.

## Data Availability

Publicly available datasets were analyzed in this study. This data can be found here: https://gwas.mrcieu.ac.uk/datasets (ID: ebi-a-GCST90018977) https://gwas.mrcieu.ac.uk/datasets (ID: ebi-a-GCST90038656).
